# Role of miR‐21‐5p/FilGAP axis in estradiol alleviating the progression of monocrotaline‐induced pulmonary hypertension

**DOI:** 10.1002/ame2.12253

**Published:** 2022-06-17

**Authors:** Xiaoyi Hu, Qian Wang, Hui Zhao, Wenhui Wu, Qinhua Zhao, Rong Jiang, Jinming Liu, Lan Wang, Ping Yuan

**Affiliations:** ^1^ Department of Cardio‐Pulmonary Circulation Shanghai Pulmonary Hospital School of Medicine Tongji University Shanghai 200433 China; ^2^ Institute of Bismuth Science University of Shanghai for Science and Technology Shanghai China

**Keywords:** 17β‐estradiol, estrogen receptor antagonist, FilGAP, miR‐21‐5p, pulmonary hypertension

## Abstract

**Background:**

Aberrant expression of microRNAs (miRNAs) has been associated with the pathogenesis of pulmonary hypertension (PH). It is, however, not clear whether miRNAs are involved in estrogen rescue of PH.

**Methods:**

Fresh plasma samples were prepared from 12 idiopathic pulmonary arterial hypertension (IPAH) patients and 12 healthy controls undergoing right heart catheterization in Shanghai Pulmonary Hospital. From each sample, 5 μg of total RNA was tagged and hybridized on microRNA microarray chips. Monocrotaline‐induced PH (MCT‐PH) male rats were treated with 17β‐estradiol (E_2_) or vehicle. Subgroups were cotreated with estrogen receptor (ER) antagonist or with antagonist of miRNA.

**Results:**

Many circulating miRNAs, including miR‐21‐5p and miR‐574‐5p, were markedly expressed in patients and of interest in predicting mean pulmonary arterial pressure elevation in patients. The expression of miR‐21‐5p in the lungs was significantly upregulated in MCT‐PH rats compared with the controls. However, miR‐574‐5p showed no difference in the lungs of MCT‐PH rats and controls. miR‐21‐5p was selected for further analysis in rats as E_2_ strongly regulated it. E_2_ decreased miR‐21‐5p expression in the lungs of MCT‐PH rats by ERβ. E_2_ reversed miR‐21‐5p target gene FilGAP downregulation in the lungs of MCT‐PH rats. The abnormal expression of RhoA, ROCK2, Rac1 and c‐Jun in the lungs of MCT‐PH rats was inhibited by E_2_ and miR‐21‐5p antagonist.

**Conclusions:**

miR‐21‐5p level was remarkably associated with PH severity in patients. Moreover, the miR‐21‐5p/FilGAP signaling pathway modulated the protective effect of E_2_ on MCT‐PH through ERβ.

## Graphical abstract

Estrogen has been shown to attenuate development of monocrotaline induced pulmonary hypertension (MCT‐PH) in rats. Aberrant expression of microRNAs (miRNAs) has been associated with the pathogenesis of pulmonary hypertension (PH). It is however not clear whether miRNAs are involved in estrogen‐rescue of PH. Our results suggest that E_2_ regulates miR‐21‐5p/FilGAP through ERβ and exerts protective effects by RhoA/ROCK2 and Rac1/c‐Jun signaling pathway to improve hemodynamic parameters and pulmonary arterial remodeling in MCT‐PH rats. This may hopefully lead to the development of novel therapeutic strategies for the management of PH.

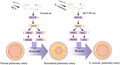

## 1 INTRODUCTION

Pulmonary hypertension (PH) is defined by an increase in pulmonary artery pressure, which causes right ventricular (RV) dysfunction and right heart failure.^1^, ^2^ The impact of sex hormones on PH has been demonstrated in several studies.^3^, ^4^, ^5^, ^6^, ^7^, ^8^, ^9^, ^10^, ^11^, ^12^, ^13^ 17β‐estradiol (E_2_) has been shown to play a protective role in hypoxia‐induced pulmonary hypertension (HPH) and monocrotaline‐induced PH (MCT‐PH) rats.^11^, ^12^, ^13^ Meanwhile, other studies have indicated that E_2_ mediates proliferation of human pulmonary artery smooth muscle cells (PASMCs) and upregulates components of the serotonin signaling system, resulting in aggravation of PH.^14^, ^15^, ^16^ Ovariectomy reversed PH in female mice with serotonin transporter overexpression.^16^ This contradictory role of sex hormones has been gaining increasing scientific interest. We have reported previously that a certain dose of E_2_ had a protective effect on MCT‐PH rats^13^ and intend to continue investigating the underlying mechanism of E_2_‐mediated prevention in PH.

Several microRNAs (miRNAs) have been identified to regulate angiogenesis and pulmonary artery (PA) remodeling in PH.^17^, ^18^, ^19^, ^20^, ^21^, ^22^, ^23^, ^24^ For example, miR‐138, miR‐143/145, and miR‐21 were significantly upregulated and miR‐204 and miR‐223 were downregulated in severe PH.^18^, ^19^, ^20^, ^22^, ^24^ miR‐21 may regulate proliferation and apoptosis of PASMCs by regulating hypoxia, inflammation, and angiogenesis signaling pathways and then coordinate pathogenic effects within the PH network.^21^, ^23^ Circulating serum miR‐21 levels have been found to be decreased in patients receiving post‐menopausal estrogen‐based hormone replacement therapies, indicating that miR‐21 is estrogen‐sensitive.^25^ The magnitude of change in circulating miRNAs levels in patients with idiopathic pulmonary arterial hypertension (IPAH) and whether the miRNAs are involved in the process of E_2_‐rescued PH remain unclear.

To further elucidate the exact role of miRNAs in E_2_‐attenuated PH, we performed microarray chips to analyze the varied expression of miRNAs in IPAH male and female patients and matched health controls and found miRNAs to be strongly regulated by E_2_ in MCT‐PH rats.

## 2 METHODS

### 2.1 Study sample

Twelve (5 males) patients with incident IPAH and 12 health controls (6 males) over the age of 18 were enrolled from Shanghai Pulmonary Hospital from May 2010 to April 2016. The new NICE clinical classification was used to make the diagnosis of IPAH.^4^ Patients with PAH due to specific causes including portal hypertension, congenital heart diseases, connective tissue diseases and PH due to left heart diseases, lung diseases, or chronic thromboembolism PH were excluded from the study. Patients suffering from acute or chronic diseases that could affect hormone metabolism (i.e., chronic autoimmune diseases, acute or chronic infections, previously diagnosed primary endocrine disorders) were also excluded, as were those receiving any hormone treatment (corticosteroids, anabolic steroids, thyroid hormones) or drugs that significantly dampen the production of hormone, either at the time of the research or previously. This research was authorized by the local ethics committee, and all patients provided written informed consent.

### 2.2 Clinical assessment

During hospitalization, demographic information, 6‐min walk distance (6MWD), body mass index (BMI), hemodynamics, N‐terminal B‐type natriuretic peptide (NT‐proBNP), and World Health Organization functional class (WHO‐FC) were assessed. Right heart catheterization (RHC) was conducted as previously described.^4^ The 6MWD test was carried out according to the ATS guidelines.^4^

### 2.3 MiRNA microarray analysis

Patients' and controls' blood samples were used to make fresh plasma. TRIzol (Invitrogen, Carlsbad, CA) was used to extract total RNA. As previously described,^26^ each sample had 5 μg total RNA tagged and hybridized on miRNA microarray chips (Exiqon Company, Denmark).

### 2.4 Animal models

After 1 week for acclimatization, adult Sprague–Dawley rats (male, ~180–200 g) were given a single subcutaneous injection of MCT (60 mg/kg, Sigma) or the same volume of 0.9% saline. On day 21, the E_2_ (75 μg/kg/day, subcutaneous, Sigma, USA), ERβ antagonist (PHTPP, 850 mg/kg/day, subcutaneous, Sigma, USA), miR‐21‐5p antagonist (AntagomiR‐21‐5p, 1 mg/kg/day, nasal drip, Biotend, China), and negative control were treated alone or in combination for about 10 days from day 22 after MCT or saline injection. Then, hemodynamics analyses and RV hypertrophy analyses were conducted as we previously described.^13^ Finally, rats were euthanized, and their lung tissues and plasma were kept at −80°C.

### 2.5 Histologic analyses and morphometry

Rat lung sections were used to perform immunostaining. The sections were treated with α‐actin antibody (Dako) to quantify PA medial wall thickness. Proliferating cells were stained with proliferating cell nuclear antigen (PCNA) staining (Dako). Apoptotic cells were observed by the TUNEL method (Apoptosis Detection Kit, Wako). The number of PCNA‐ and TUNEL‐positive cells in 10 fields of each lung section was estimated as a proportion of the total cell number at a magnification of 400 in a blinded manner.^13^, ^27^ Quantification of PA medial wall thickness was conducted by counting α‐actin (+) vessels per field of view at a magnification of 400.^13^, ^27^ Eight small pulmonary vessels of each animal with an outer diameter ranging from 10 to 50 μm were evaluated.^13^, ^28^

### 2.6 miRNA transfection and luciferase assay

DNA fragments of FilGAP mRNA 3′ untranslated region (UTR) bearing the probable miR‐21‐5p binding sequence were synthesized by Invitrogen. Then, these fragments were cloned in the pMIR‐REPORTTM luciferase miRNA expression reporter vector (Ambion, USA) at the multiple cloning sites downstream of the luciferase gene (Hind III and SpeI sites). HEK293 cells (1–10^5^ per well) were transfected with 0.1 μg PRL‐TK (TK‐driven Renilla luciferase expression vector) and 1 μg miR‐21‐5p or 1 μg PGL3‐target DNA (firefly luciferase vector) with Lipofectamine 2000 (Invitrogen, USA) after 24 hours in serum‐free medium. A dual luciferase reporter assay kit (Promega, USA) was used to quantify luciferase activities 48 hours after transfection using a luminometer (LumatLB9507, USA).^24^

### 2.7 Quantification of mRNA and miRNA levels

Lung tissues were lysed in TRIzol reagent, and total RNA was isolated. The protocol for synthesis of cDNA is as previously described.^29^ For the measurement of FilGAP mRNA, conventional RT‐qPCR was adopted. For quantification of miRNAs, the mirVana qRT‐PCR miRNA Detection Kit (Ambion, USA) was used according to the methods described elsewhere.^19^, ^30^

### 2.8 Western blot analyses

The protein lysates were taken from lung tissues, with the protocol previously described.^13^ SDS‐PAGE was used to fractionate protein samples (50 μg). FilGAP (Abcam, USA), RhoA (Cell Signaling Technologies [CST], USA), ROCK2 (CST, USA), Rac1 (CST, USA), and c‐Jun (CST, USA) were used as primary antibodies, with β‐actin (Abcam, USA) as an internal control.

### 2.9 Statistical analyses

The clinical results were presented as absolute numbers for categorical variables and mean with standard deviation (SD) or median (with interquartile range) for continuous variables. For continuous variables, the *t*‐test or Mann–Whitney *U* test were used to make comparisons. The Spearman rho coefficient was used to examine correlations. Univariate linear analysis with forward/backward multiple stepwise linear regression analysis with hemodynamic variables as the dependent outcome was performed to determine the strength of the association between hemodynamics and miRNAs, and correlation with IPAH was found. To adjust multiple regression analysis, age, BMI, and WHO‐FC were forced into the models.

The basic results from multiple experiments were presented as mean with standard error. Student's *t*‐tests were used to compare 2 groups, and one‐way analysis of variance (ANOVA) was used to compare multiple groups. Statistical significance was defined as *p* < .05. GraphPad Prism (version 9.0.1) and Figdraw (www.figdraw.com) were used to plot figures.

## 3 RESULTS

### 3.1 Circulating miRNAs in patients with IPAH and controls

Demographics, baseline, and hemodynamic data are summarized in Table 1. There were no differences in age, heart rate, blood pressure, and BMI between patients with IPAH and controls. Patients with IPAH had higher mean right atrial pressure (mRAP), mean pulmonary arterial pressure (mPAP), mean pulmonary arterial wedge pressure (mPAWP), and pulmonary vascular resistance (PVR), and lower cardiac output (CO) and cardiac index (CI), at diagnosis.

**TABLE 1:** Baseline characteristics in patients with IPAH and control groups

	Patients with IPAH (*n* = 12)	Controls (*n* = 12)	*p*‐value
*Baseline characteristics*			
Age, years	36.3 ± 1.9	36.6 ± 1.6	.718
Male/female, *N*	5/7	6/6	.698
HR, bpm	83.5 ± 17.4	75.4 ± 14.5	.667
SBP, mm Hg	113.0 ± 15.5	124.5 ± 17.9	.118
DBP, mm Hg	71.4 ± 11.8	74.9 ± 10.2	.530
BMI, kg/m^2^	23.8 ± 2.5	22.4 ± 3.3	.702
6MWD, m	378.9 ± 84.8	–	–
NT pro‐BNP, pg/ml	287 (203, 661)	–	–
WHO‐FC, *n* (%)			
I–II	4 (33.3)	–	–
III–IV	8 (66.7)	–	–
*Hemodynamics*			
mRAP, mm Hg	6.3 ± 5.4	2.0 ± 1.9	.026
mPAP, mm Hg	68.8 ± 22.0	12.8 ± 2.9	<.001
mPAWP, mm Hg	9.1 ± 3.7	5.4 ± 2.3	.013
PVR, Wood units	15.6 ± 6.8	1.1 ± 0.6	<.001
CO, L/min	4.2 ± 1.6	7.0 ± 1.8	.001
CI, L/min/m^2^	2.6 ± 1.1	4.2 ± 1.1	.003
*Specific medications*			
PDE‐5 inhibitors, %	4 (33.3)	–	–
ERAs, %	3 (25.0)	–	–
Prostacyclin analogs, %	2 (16.7)	–	–
Combination, %	2 (16.7)	–	–
Nonspecific medication, %	1 (8.3)	–	–

*Note*: Comparisons were performed using *t*‐test or Mann–Whitney *U* test for continuous variables.

Abbreviations: 6MWD, 6‐minute walk distance; BMI, body mass index; CI, cardiac index; CO, cardiac output; DBP, diastolic blood pressure; ERA, endothelial receptor antagonist; HR, heart rate; mPAP, mean pulmonary arterial pressure; mPAW, mean pulmonary arterial wedge pressure; mRAP, mean right atrial pressure; NT pro‐BNP, N‐terminal pro‐brain natriuretic peptide; PDE‐5, phosphodiesterase type 5; PVR, pulmonary vascular resistance; SBP, systolic blood pressure; WHO‐FC, World Health Organization Functional Class.

**FIGURE 1:**
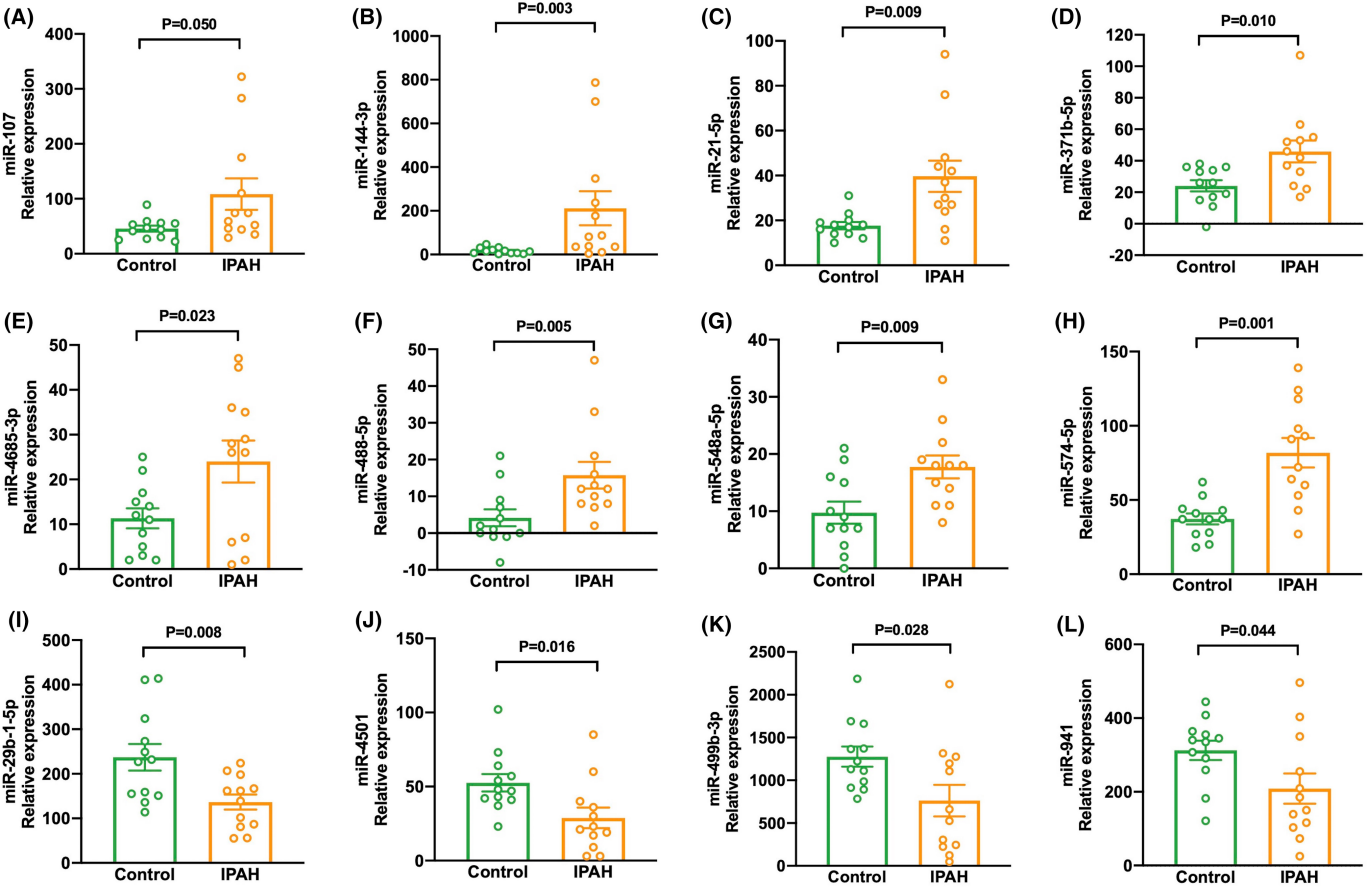
Differences of selected miRNAs in plasma samples of patients with IPAH and controls. (A–L) Fluorescence values of miR‐107, miR‐144‐3p, miR‐21‐5p, miR‐371b‐5p, miR‐4685‐3p, miR‐488‐5p, miR‐548a‐5p, miR‐574‐5p, miR‐29b‐1‐5p, miR‐4501, miR‐499b‐3p, and miR‐941 in patients with IPAH and controls.

Circulating miR‐107, miR‐144‐3p, miR‐21‐5p, miR‐371b‐5p, miR‐4685‐3p, miR‐488‐5p, miR‐548a‐5p, and miR‐574‐5p were markedly expressed in patients, while miR‐29b‐1‐5p, miR‐4501, miR‐499b‐3p, and miR‐941 levels were significantly downregulated (Figure 1).

### 3.2 Sex differences of miRNAs in patients with IPAH and controls

**FIGURE 2:**
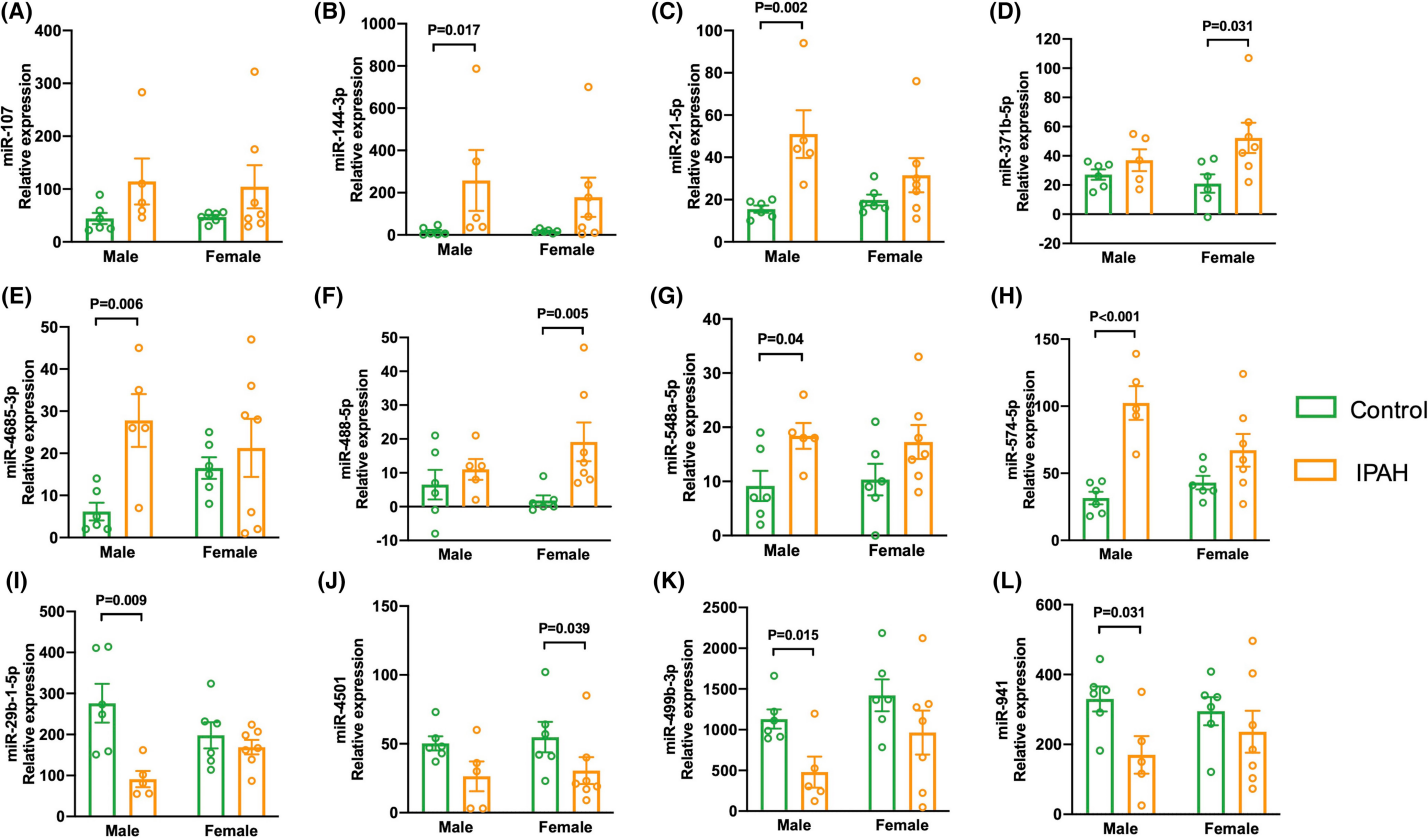
Sex differences in the expression of selected miRNAs in plasma samples of patients with IPAH and controls. (A–L) Sex differences in the fluorescence values of miR‐107, miR‐144‐3p, miR‐21‐5p, miR‐371b‐5p, miR‐4685‐3p, miR‐488‐5p, miR‐548a‐5p, miR‐574‐5p, miR‐29b‐1‐5p, miR‐4501, miR‐499b‐3p, and miR‐941 in patients and controls.

Subsequently, we found sex differences in the expression of aforementioned miRNAs in plasma samples of patients with IPAH (Figure 2). miR‐144‐3p, miR‐21‐5p, miR‐4685‐3p, miR‐548a‐5p, miR‐574‐5p, miR‐29b‐1‐5p, miR‐499b‐3p, and miR‐941 levels showed significant differences between male patients and controls. miR‐371b‐5p, miR‐488‐5p, and miR‐4501 levels showed significant differences between female patients and controls. Of note, miR‐144‐3p, miR‐21‐5p, and miR‐574‐5p levels were remarkably higher in male patients than in male controls, but these miRNAs showed no differences between female patients and controls.

### 3.3 Circulating miRNAs associated with hemodynamic parameters in patients with IPAH

Correlations between circulating miRNAs and hemodynamic parameters are summarized in Table 2. Circulating miR‐144‐3p, miR‐21‐5p, and miR‐574‐5p fluorescence values had significant positive correlation with mPAP in subjects. To determine the strength of each individual parameter to predict mPAP elevation, variables with significant correlations were entered into univariate linear analysis and stepwise multiple linear regression analyses. As shown in Table 3, circulating miR‐21‐5p and miR‐574‐5p levels were found to be the independent predictors of mPAP elevation and accounted for 76.5% (*R* ^2^ = .765) of the variation.

**TABLE 2:** The correlations between mPAP and miRNAs

	*r*	*p*‐value
miR‐144‐3p	.658	.001
miR‐21‐5p	.680	<.001
miR‐574‐5p	.826	<.001

*Note*: Correlations were assessed using rho coefficient of Spearman.

Abbreviation: mPAP, mean pulmonary arterial pressure.

**TABLE 3:** Determinants of mPAP in all patients with IPAH

Dependent variables	Independent variables	Univariate analysis			Multiple analysis^a^		
		*R* ^2^	95% CI	*p*‐value	*R* ^2^	95% CI	*p*‐value
mPAP	miR‐144‐3p	.478	0.054 to 0.153	<.001	–	–	–
	miR‐21‐5p	.492	0.794 to 2.152	<.001	.765	−3.577 to −0.425	.015
	miR‐574‐5p	.668	0.608 to 1.152	<.001	.765	1.048 to 2.647	<.001

*Note*: Univariate linear analysis with forward/backward multiple stepwise linear regression analysis was performed.

Abbreviations: CI, confidence interval; mPAP, mean pulmonary arterial pressure.

^a^ Adjusted by age, sex, and BMI.

### 3.4 Improvement of MCT‐PH rats by E_2_ and ERβ

**FIGURE 3:**
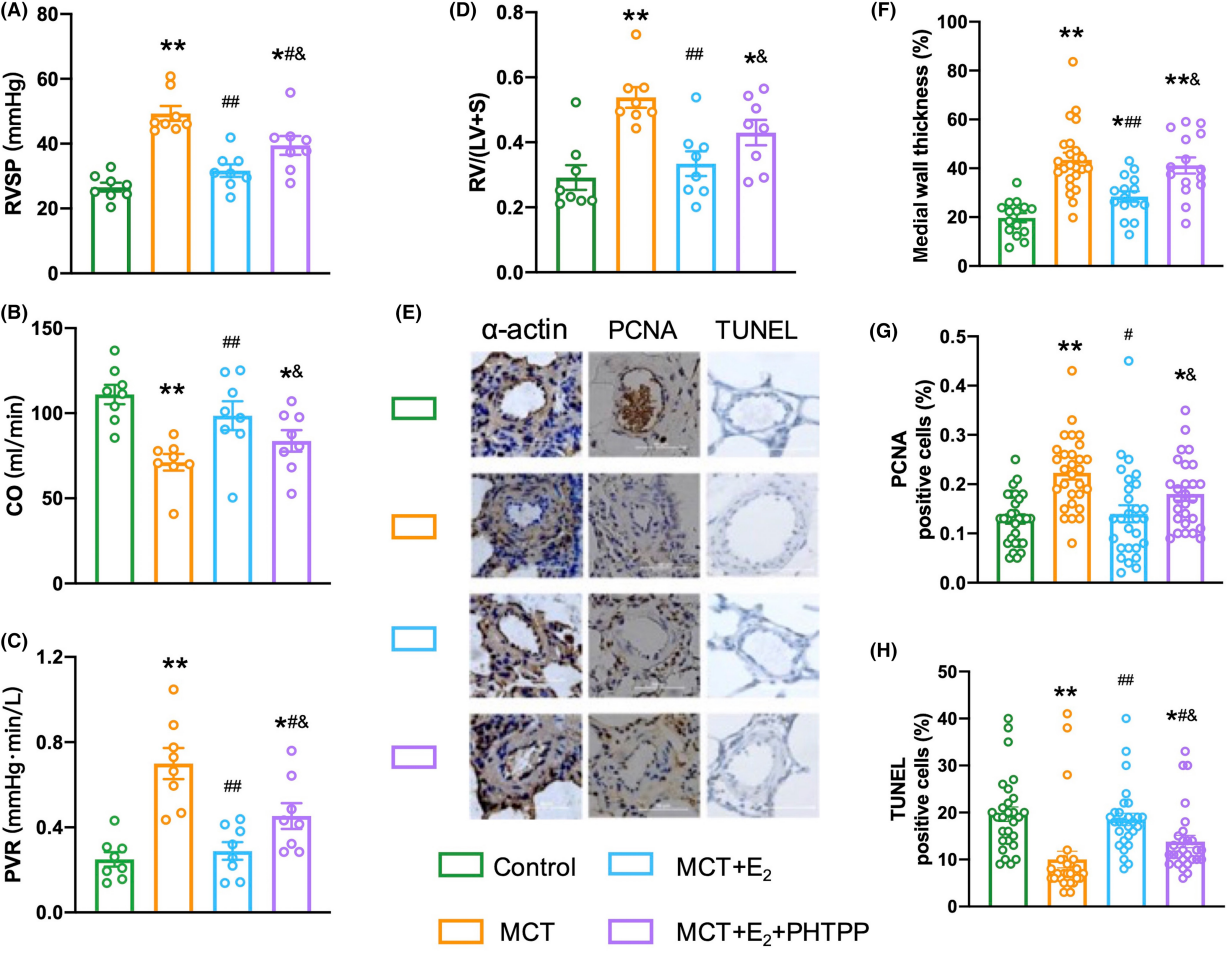
E_2_ improved MCT‐PH rats by targeting ERβ. (A–D) Changes of RVSP, CO, PVR, and RV/(LV + S) weight ratio in all groups. (E) Histology images of pulmonary arteries in the groups. (F–H) The degree of medial wall thickness, PCNA‐positive cells, and TUNEL‐positive cells of small pulmonary arteries in all groups. *p < .05, **p < .01 versus control rats; ^#^ *p* < .05 and ^##^ *p* < .01 versus MCT‐PH rats; ^&^ *p* < .05 and ^&&^ *p* < .01 versus E_2_ in MCT‐PH rats.

In MCT‐PH rats, E_2_ treatment significantly decreased RV systolic pressure (RVSP), PVR, and RV/(LV + S) weight ratio, and increased CO compared with control groups (Figure 3A‐D). PHTPP dramatically reversed the above effects of E_2_ in MCT‐PH rats (Figure 3A–D). Treatment with E_2_ also inhibited pulmonary arterial medial wall thickness, PCNA expression and enhanced TUNEL‐positive cells in MCT‐PH rats in an ERβ‐dependent manner (Figure 3E–H)

### 3.5 Regulating miR‐21‐5p expression by E_2_ and ERβ in MCT‐PH rats

**FIGURE 4:**
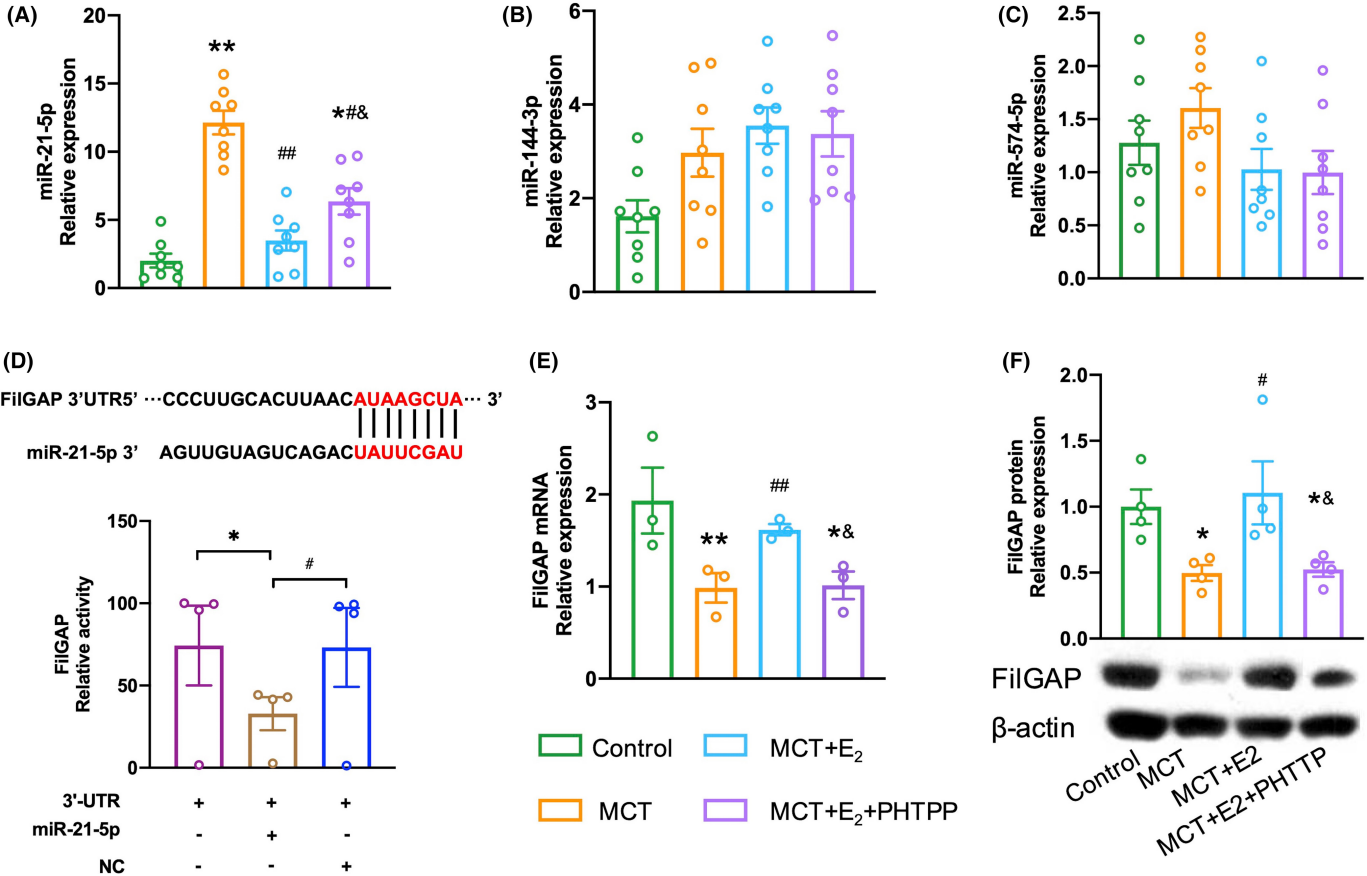
E_2_ regulated miR‐21‐5p and FilGAP by ERβ in MCT‐PH rats. (A–C) The effect on expression of selected miRNAs regulated by E_2_/ERβ. (D) FilGAP verified as target of miR‐21‐5p in HEK293 cells. Changes in luciferase reporter activities show the interaction between miR‐21‐5p and FilGAP 3′ UTR. (E,F) E_2_ reversed the downregulation of FilGAP by targeting ERβ in lung of rats with PH. *p < .05, **p < .01 versus control rats (or 3′ UTR); ^#^ *p* < .05, ^##^ *p* < .01 versus MCT‐PH rats (or 3′ UTR + miR‐21‐5p); ^&^ *p* < .05 versus E_2_ in MCT‐PH rats.

Similar to what was observed in human patients with IPAH, the expression of miR‐21‐5p in the lungs was remarkably increased in MCT‐PH rats compared with the controls. However, miR‐144‐3p and miR‐574‐5p showed no difference in the lungs of MCT‐PH rats and controls (Figure 4A–C). In addition, E_2_ treatment markedly decreased miR‐21‐5p expression in MCT‐PH rats (Figure 4A). Treatment with E_2_ in combination with PHTPP increased miR‐21‐5p expression significantly compared with E_2_ alone in MCT‐PH rats (Figure 4A).

### 3.6 Modulating expression of miR‐21‐5p and targeting downstream signaling pathways by E_2_ and ERβ in MCT‐PH rats

The TargetScan software (http://www.targetscan.org) suggested that FilGAP was one of the highly conserved predicted targets of miR‐21‐5p (Figure 4D). The luciferase activity assay determined that FilGAP targets miR‐21‐5p as transfection of miR‐21‐5p significantly decreased the relative luciferase activity in HEK 293 cells (Figure 4D).

Compared with controls, expression of FilGAP mRNA and protein was significantly reduced in the lungs of MCT‐PH rats (Figure 4E,F). FilGAP was effectively restored by E_2_ administration at both mRNA and protein level. Addition of PHTPP could attenuate the protective effects of E_2_ in MCT‐PH rats (Figure 4E,F).

**FIGURE 5:**
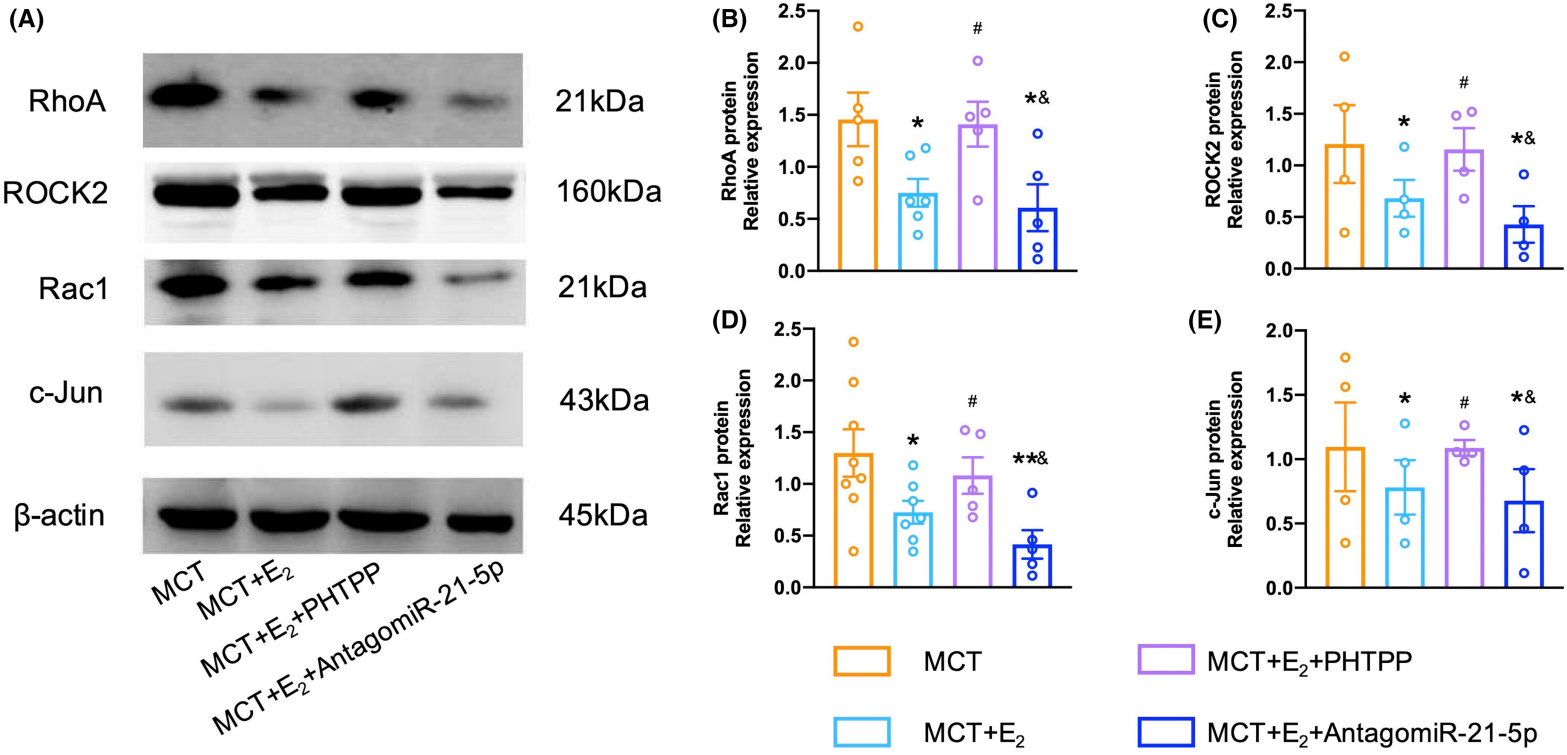
E_2_ Regulated downstream signaling pathways of miR‐21‐5p by ERβ in MCT‐PH rats. (A) Representative immunoblots of lung lysates with anti‐RhoA, anti‐ROCK2, anti‐Rac1, anti‐c‐Jun, and anti‐β‐Actin in rats. (B–E) E_2_ significantly regulated pulmonary RhoA, ROCK2, Rac1, and c‐Jun expressions by targeting ERβ or miR‐21‐5p antagonist in MCT‐PH rats. *p < .05, **p < .01 versus MCT‐PH rats; ^#^ *p* < .05 versus E_2_ in MCT‐PH rats. ^&^ *p* < .05 versus E_2_ + PHTPP in MCT‐PH rats.

As shown in Figure 5, compared with the MCT‐PH rats, E_2_ treatment significantly inhibited the expressions of RhoA, ROCK2, Rac1, and c‐Jun proteins. PTHPP markedly weakened the effects of E_2_ in MCT‐PH rats (Figure 5). In addition, E_2_ administered in combination with miR‐21‐5p antagonist downregulated RhoA, ROCK2, Rac1, and c‐Jun expressions compared with untreated MCT‐PH rats (Figure 5).

## 4 DISCUSSION

Sex differences of miRNAs have received far less attention in IPAH. To the best of our knowledge, the present investigation is the first to report miRNAs as predictors of mPAP change in IPAH. We explored whether E_2_ could affect the expression of these miRNAs. Our data indicate that E_2_ downregulated miR‐21‐5p expression in addition to regulating its downstream signaling pathways, RhoA/ROCK2 and Rac1/c‐Jun, with subsequent improvement in PA remodeling by targeting ERβ in PH rats.

Despite our small sample size, we noted significant differences in hemodynamics between patients with IPAH and controls at the time of diagnosis. We observed differential expression of miRNAs between the 2 groups. To explore the relationship between miRNAs and hemodynamics, we performed linear regression analysis, which showed that several miRNAs, including miR‐21‐5p and miR‐574‐5p, were independent predictors of mPAP. These results further supported the critical role of miRNAs in the development of IPAH. Based on the foundation of our previous research^29^ regarding the protective effect of estrogen in PH, albeit not a definite conclusion, we further detected some sex differences regarding miRNAs. Our data revealed several miRNAs with significant sex differences between IPAH and controls. Thus, we speculated that these miRNAs may have a participatory role in conjunction with estrogen in IPAH.

The term “estrogen paradox” appears in PAH since it is seen more frequently in women with PH despite better mRAP and mPAP at diagnosis and better survival compared with men.^3^, ^4^, ^5^, ^6^, ^7^, ^31^ E_2_ administration improved hemodynamic parameters, suppressed PA remodeling and RV hypertrophy in female MCT‐PH rats, validated by our earlier research regarding the role of estrogen in PH.^13^ The present study confirmed that E_2_ ameliorated MCT‐PH in male rats. These findings were consistent with the results by Umar et al.^32^ Yet, our findings contradict the findings of Tofovic et al.,^12^, ^33^ who reported that the beneficial effects of E_2_ in MCT‐PH rats were mediated by its downstream metabolite 2‐methoxyestradiol (2‐ME) as 2‐ME could attenuate the development of MCT‐PH.^12^, ^33^ Furthermore, both E_2_ and 2‐ME could increase prostacyclin and NO release, inhibit endothelin synthesis, and improve vascular remodeling by phosphorylated Akt pathway in MCT‐PH.^12^, ^13^ Certainly, there is evidence supporting that E_2_ has consistent positive effects in the setting of hypoxic pulmonary vasoconstriction and HPH,^11^, ^34^, ^35^, ^36^ whereas no effects were seen in PH models of BMPR2 mutants and serotonin upregulation.^15^, ^37^ Therefore, E_2_ effects may be highly “context specific” and should be seen as a double‐edged sword in PH.

Multiple animal studies demonstrate that E_2_ affects the pathogenesis of PH possibly by regulating genetic factors, growth factors, cytokines, and other environmental stressors. However, it remains unclear whether the miRNAs are involved in the process of E_2_ rescuing MCT‐PH. Several miRNAs are known to be altered by PH.^17^, ^18^, ^19^, ^20^, ^21^, ^22^, ^23^, ^24^, ^26^ We chose to investigate whether miR‐21‐5p was the key to understanding effects of E_2_ in MCT‐PH, as miR‐21‐5p was altered in HPH.^19^, ^22^, ^23^ Our data indicate that E_2_ downregulated the pulmonary expression of miR‐21‐5p in MCT‐PH rats. This was similar to the results of Kangas et al., who reported miR‐21‐5p levels to be lower in estrogen‐sensitive women with postmenopausal estrogen‐based hormone replacement therapy.^25^ Casaburi et al.^38^ reported that androgen, the synthetic substrate of E_2_, downregulated miR‐21 overexpression in breast cancer. Thus, we indicated that miR‐21‐5p was involved in the protective effects of E_2_ in MCT‐PH.

We chose to explore the above proteins and downstream signaling pathways for 2 main reasons. Firstly, luciferase activity assay indicated that FilGAP, also known as Arhgap 24, was a target of miR‐21‐5p, which was also examined in the study of Meng et al.^39^ However, until now, there have been few studies on FilGAP in PH rats. Secondly, FilGAP is a Rho GTPase‐activating protein. Previous studies have indicated that small GTPases (Rho family) regulate a variety of key cellular functions, including PASMCs cell adhesion, migration, proliferation, vesicle trafficking, and differentiation.^40^, ^41^, ^42^ Thus, FilGAP functions as a mediator in Rho/ROCK‐dependent cell biology activities such as lamellipodia formation, cell spreading, adhesion, migration, and membrane protrusion.^34^, ^43^, ^44^ Strikingly, E_2_ increased FilGAP expression and inhibited the downstream signaling pathways RhoA, ROCK2, Rac1, and c‐Jun. Many studies have revealed that RhoA/ROCK, Rac1/c‐Jun signaling pathways promote PASMCs proliferation and PA remodeling in PH.^45^, ^46^ Therefore, our data demonstrate that E_2_ plays a wide‐ranging role in regulating miR‐21‐5p/FilGAP and the downstream signaling pathways (RhoA/ROCK2 and Rac1/c‐Jun) to impact other cell biology activities in MCT‐PH rats.

E_2_ exerts its biological effects primarily through ERα or ERβ in different PH models.^32^, ^47^ The present study indicated that treatment with ERβ antagonists attenuates the protective effects of E_2_ on hemodynamic alterations, PA remodeling, and RV hypertrophy in MCT‐PH. Umar and colleagues also indicated that E_2_ rescued PH predominantly by regulating ERβ.^32^ They used ERβ agonist (DPN) and ERβ antagonist (PHTPP) treatment to confirm that E_2_‐mediated protections are regulated through ERβ, and ERα agonist and combined treatment failed to rescue MCT‐PH. They did not apply ERα antagonist (MPP) to investigate its impact on hemodynamic alterations. Lahm and colleagues^47^ suggested that the considerable effect of E_2_ on functional endpoints in HPH was mostly mediated by ERα. This discrepancy could be due to different E_2_ signaling systems in HPH compared with those exposed to MCT. However, according to Lahm's study, the importance of ERβ in HPH should not be overlooked, as there were trends that suggested a partial or minor contribution of ERβ to E_2_ signaling in the right ventricular capillary/myocyte ratio in addition to attenuation of inhibitory effects of E_2_ on PA remodeling and ERK1/2 activation in hypoxic pulmonary artery endothelial cells after treatment with ERβ antagonist.^47^ Of note, although ERβ antagonist significantly attenuated E_2_ effects on miR‐21‐5p expression, there was striking inhibition effects of E_2_ on the downstream signaling pathways through ERβ blockade in our study. Thus, we believe that E_2_ may act through a similar ERβ‐mediated pathway to rescue MCT‐PH.

## 5 CONCLUSION

**FIGURE 6:**
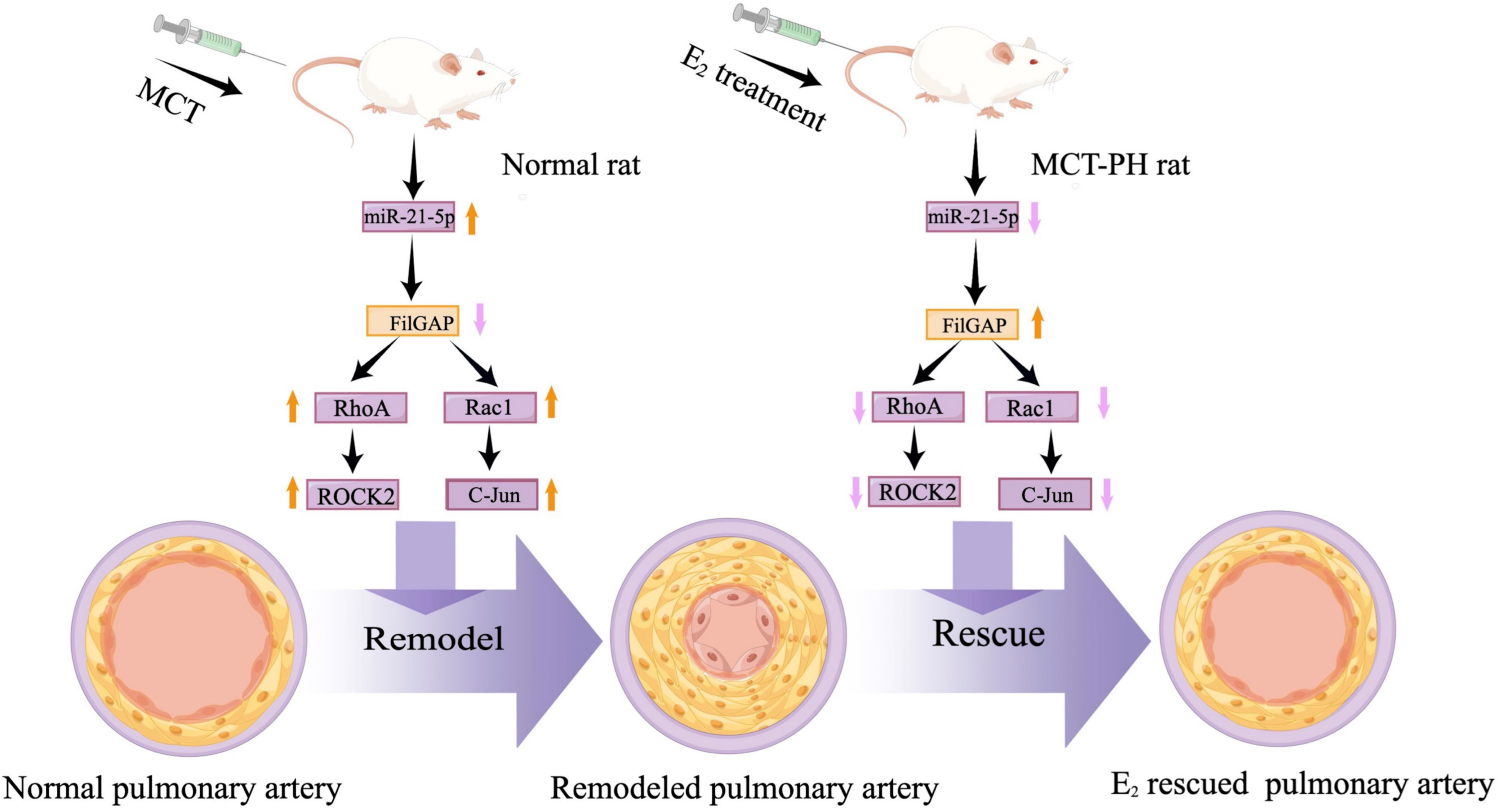
Schematic diagram showing inhibition of miR‐21‐5p/FilGAP axis and rescue of pulmonary remodeling by E_2_.

In conclusion, our data suggest that E_2_ through ERβ exerts protective effects by regulating the miR‐21‐5p/FilGAP signaling pathway to improve hemodynamic parameters and pulmonary arterial remodeling in MCT‐PH rats (Figure 6). This may lead to the development of novel therapeutic strategies for the management of PH.

## Ethics approval

This study complied with the Declaration of Helsinki (as revised in 2013) and was approved by the Medical Ethics Committee of Shanghai Pulmonary Hospital. Animal experiments were authorized by the Institutional Committee for Use and Care of Laboratory Animals of Tongji University (Shanghai, China).

## CONFLICT OF INTEREST

The authors have no conflicts of interest.

## AUTHOR CONTRIBUTIONS

P.Y. conceived the project and supervised the research. X.H., Q.W. and H.Z. designed all the experiments, performed the experiments, and analyzed the data with the assistance of W.W., Q.Z., R.J., J.L., and L.W. Y.M. and X.H. wrote the manuscript, data analysis, and paper discussion.

## PATIENT CONSENT STATEMENT

All patients have submitted informed consents.

## Data Availability

The raw data supporting the conclusions of this article will be made available by the authors, without undue reservation.
